# Red blood cells and their releasates compromise bone marrow-derived human mesenchymal stem/stromal cell survival in vitro

**DOI:** 10.1186/s13287-021-02610-4

**Published:** 2021-10-21

**Authors:** Ryan Christopher Dregalla, Jessica Ann Herrera, Edward Jeffery Donner

**Affiliations:** 1 4795 Larimer Parkway, Elite Regenerative Stem Cell Specialists, LLC, Johnstown, CO 80534 USA; 2 R&D Regenerative Laboratory Resources, LLC, 4795 Larimer Parkway, Johnstown, CO 80534 USA; 3 4795 Larimer Parkway, Colorado Spine Institute, PLLC, Johnstown, CO 80534 USA

**Keywords:** Mesenchymal stem cells, Bone marrow aspirate concentrate, Hematocrit, Red blood cells, Viability, Apoptosis, Necrosis

## Abstract

**Purpose:**

The use of bone marrow aspirate (BMA) and bone marrow aspirate concentrate (BMC) in the treatment of inflammatory orthopedic conditions has become a common practice. The therapeutic effect of BMA/BMC is thought to revolve primarily around the mesenchymal stem/stromal cell (MSC) population residing within the nucleated cell fraction. MSCs have the unique ability to respond to site of injury via the secretion of immunomodulating factors, resolving inflammation in diseased joints. Recently, the importance of hematocrit (HCT) in BMC has been debated, as the potential impact on MSC function is unknown. In the present study, we investigate MSC health over a short time-course following exposure to a range of HCT and red blood cell releasate (RBC_rel_) conditions.

**Methods:**

Bone marrow-derived human MSCs in early passage were grown under conditions of 0%, 2.5%, 5%, 10%, 20% and 40% HCT and RBC_rel_ conditions for 3 days. At each day, the percentage of viable, apoptotic and necrotic MSCs was determined via flow cytometry. Relative viable MSC counts in each condition was determined to account for dynamic changes in overall MSC densities over the time-course. Statistical analysis was performed using a one-way ANOVA comparing test conditions to the control followed by a Dunnett’s multiple comparison test.

**Results:**

Significant reductions in viable MSCs concurrent with an increase in necrotic MSCs in high HCT and RBC_rel_ conditions was observed within 24 h. At each successive timepoint, the percent and relative number of viable MSCs were reduced, becoming significant in multiple HCT and RBC_rel_ conditions by Day 3. Necrosis appears to be the initial mode of MSC death following exposure to HCT and RBC_rel_, followed by apoptosis in surviving MSC fractions.

**Conclusion:**

Various levels of HCT and RBC_rel_ severely compromise MSC health within 3 days and HCT should be controlled in the preparation of BMC products. Further, HCT of BMCs should be routinely recorded and tracked with patient outcomes along with routine metrics (e.g. nucleated cell counts, fibroblast-colony forming units). Differences in HCT may account for the inconsistencies in the efficacy of BMC reported when treating orthopedic conditions.

**Supplementary Information:**

The online version contains supplementary material available at 10.1186/s13287-021-02610-4.

## Background

As the field of regenerative medicine grows in popularity, the number of medical devices available to process orthobiologics continues to expand, each claiming an array of potential benefits. To date, the most routine autologous orthobiologics produced include platelet-rich plasma (PRP), bone marrow aspirate/bone marrow aspirate concentrate (BMA/BMC) and micronized adipose tissue (MFat). All technologies and protocols used to generate these products result in an injectable format, allowing for minimally invasive approaches to treating orthopedic and spinal conditions. As the popularity of these therapeutics increase, important observations have been made illustrating innate differences between final products derived in the same classification (e.g. PRP, BMC) which may influence the applicability and efficacy of the treatment for a specific condition. As an example, in PRP formulations, an orthobiologic used for its growth factor and bioactive protein content [1], the final concentration or increase in platelet count per unit volume over the whole blood baseline is important. While in certain conditions, intermediate-to-high concentrations of platelets are favorable [2], hyper-concentration may have deleterious effects in certain cases [2–4] indicating the importance of orthobiologic dose per musculoskeletal (MSK) condition. Further, the concentration of leukocytes in the final product has been shown to be a determining factor in the suitability of the product for a given condition [5]; higher leukocyte concentrations may be useful in the treatment of ligament and tendons [6] whereas leukocyte poor/depleted PRP may be more useful in mitigating inflammation and stimulating resident cells in cartilage-like tissue [7, 8]. The scope of this work is expanding and is essential in, understanding the molecular bases for these products and optimizing clinical outcomes.

The rationale for the use of BMA/BMC therapeutically is owed to the presence of mesenchymal stem/stromal cells (MSCs) which have the ability to secrete immunomodulatory and trophic factors [9, 10]. While the presence of MSCs in bone marrow is well documented, they are a relatively rare population amongst the total nucleated cell count (TNC), accounting for 0.001—0.01% of the TNC [11] or less [12]. Similar to investigating the benefits and negative effects of platelet concentration in PRP, BMA/BMC therapies are highly focused on TNC and colony forming units with fibroblast morphology (CFU-f) in vitro as a proxy for MSC quantitation [13]. While debatable, the rationale for using these metrics is that increasing TNC will result in higher MSC-derived CFU-fs which, may correlate with patient outcomes [14, 15] due to the anti-inflammatory nature of the MSCs [16]. Collectively, processing methods and devices focus on maximizing TNC and MSC-derived CFU-fs. Typically, this approach results in increasing hematocrit (HCT), as a percentage of MSCs reside slightly below the buffy coat-red blood cell interface following centrifugation.

While cultured MSC surface markers are designated by the International Society for Cellular Therapy as expressing CD73, CD90, CD105 and lack CD45, CD34, CD14 or CD11b, CD79a or CD19 and HLA class II, MSC subtypes within the bone marrow niche have been identified using different markers [17]. Various combinations of the surface markers CD271, CD146 and Leptin receptor along with intracellular labeling of Nestin and CXCL12-expressing (CXCL12-abundant reticular (CAR)) MSCs provide insight to their location in the bone marrow compartment [18, 19] and specific roles in vivo [20]. Importantly, each of these MSC subtypes in the bone marrow has a crucial role in regulating hematopoietic cell function [18, 21, 22], which may have additional benefits in immunomodulation. As the therapeutic value of BMC may be reliant on a variety of different MSC subtypes, ensuring MSC health in vivo is essential to optimize clinical outcomes.

Recently, there has been a growing interest in understanding the impact of erythrocytes/red blood cells (RBCs) in orthobiologic products. Researchers as well as manufacturers of commercially available orthobiologic kits are routinely reporting HCT which is perceived as an important but uncharacterized metric [23], particularly for BMC. In a recent review by Everts et al., the various potential molecular bases for RBC toxicity and pro-inflammatory processes in MSK regenerative therapies was thoroughly described [24]. This review provides an explanation for the results of studies highlighting the negative impact of RBC/HCT on resident cell of orthopedic tissues, including synoviocytes [25] and chondrocytes [26]. However, no research has been done to investigate the impact of RBCs/HCT on MSC health and if there is a translational value to this metric.

In the present study, we aim to investigate the impact of HCT and factors released from RBCs (releasates) on cultured MSCs in vitro. RBCs were collected from residual BMA and added to MSC culture at 2.5%, 5%, 10%, 20% and 40% HCT and compared to the 0% control containing culture media only. At Days 1, 2 and 3, MSC cultures were assayed for viability via fluorescent microscopy and flow cytometry. As RBCs release various factors within days under in vitro conditions which are potentially inflammatory [27] and cytotoxic [24], RBC solutions were generated and aged at 37˚C for 72 h to produce RBC releasates (RBC_rel_) for each respective condition. MSC cell cultures were then exposed to RBC_rel_ and assayed at Days 1, 2 and 3. Our results clearly demonstrate a deleterious effect of both RBCs based on HCT and their releasates on MSC health in vitro, highlighting the importance of controlling this variable in the formulation of orthobiologics where MSCs are of interest.

## Methods

### Obtaining bone marrow-derived RBCs

Patients scheduled for a bone marrow aspirate concentrate treatment provided informed consent for the use of residual and waste products for research. Following the procedure, residual red blood cells were retained and processed according to the procedure below. Data represents RBCs from 5 donors ranging from 48 to 74 years of age.

### Preparation of HCTs for MSC culture

Plasma and buffy coat were removed in the production of BMC for patient use. The residual bone marrow aspirate, devoid of the buffy coat and plasma, was centrifuged at 500 relative centrifugal force (RCF) for 5 min. Remaining plasma and nucleated cells (NC) were removed along with an additional 2 milliters (mL) of RBCs to create packed RBCs. RBCs were then mixed with total DMEM (10% Fetal Bovine Serum (FBS) (Peak Serum, cat #: PS-FB3) in Dulbecco’s Modified Eagle Medium (DMEM) (Gibco, cat #: 10567–014)) to create stock HCT solutions at 5%, 10%, 20%, 40% and 80% by volume (e.g. 80% HCT is 40 mL packed RBCs + 10 mL total media). In culture, these stocks were diluted 1:1 in total media to create final HCTs of 2.5%, 5%, 10%, 20%, and 40%. The 0% control was total media alone. A single RBC donor was used for each experimental series, RBCs were not pooled.

### Production of RBC_rel_ for MSC culture

RBC_rel_ was created by adding red blood cells (RBCs) to total media as described above, resulting in final HCTs of 5%, 10%, 20%, 40%, and 80% by volume. The HCT solutions were produced to allow for the supernatant to have at least 10 mL of the media component (e.g. 80% HCT contained 40 mL packed RBCs and 10 mL total media). HCT solutions were incubated at 37˚C at 5% CO_2_ for 3 days to allow for RBC degradation and release of associated factors. After the 3-day incubation, preparations were centrifuged at 500 RCF for 5 min to separate RBCs. To minimize the collection of RBCs, 90% of the media component added to the HCT preparation was collected from each RBC_rel_ and stored at 4˚C (e.g. of the 10 mL of media in the 80% HCT preparation, 9 mL media was collected). Within 24 h, RBC_rel_ were diluted 1:1 in total media and added to MSC cultures resulting in the final 2.5%, 5%, 10%, 20%, and 40% RBC_rel_ conditions. The 0% control consisted of total media only.

### Establishment of MSC cultures for HCT and RBC_rel_ assays

Bone marrow-derived MSCs were recovered from cryogenic containment and seeded at 500,000 cells per T-75 cell culture flask (CellTreat cat #: 229341) in total media and cultured for 3 days. MSCs were passaged and prepared for HCT and RBC_rel_ exposure by seeding 6-well plates or T-25 s. For fluorescent microscopy assays, 20,000 MSCs per well in a 6 well plate (CellTreat, cat #: 229106) for each respective condition. One plate was established for each time point. For flow cytometry analysis, 62,500 MSCs per T-25 (CellTreat, cat #: 229331) were seeded for each respective condition and each time point (7 flasks per time point, allowing for an isotype and dye control). Cells were allowed to adhere for 24 h in total media at 37˚C, 5% CO_2_. After 24 h, either HCTs or RBC_rel_ was added to all cultures in the experimental series. A 6-well plate or series of T-25 s for HCT and RBC_rel_ experiments were assessed at Days 1, 2 and 3. MSCs used in experiments were between passages 3 and 5 and were not matched with RBC donors. All HCT and RBC_rel_ experimental series were repeated twice in fluorescent microscopy assays and three times in flow cytometry assays without intra-donor replicates.

### Collection and preparation of MSCs from culture

Initially, 2 mL of phosphate buffered saline (PBS) (Gibco cat #: 10010023) was added to each well or flask and incubated at room temperature on an orbital shaker for 2 min. All media was collected in a 15 mL conical (Cell Treat, cat #: 229411) for each condition. Flask or well was then washed twice with 2 mL of PBS. Each wash was retained and pooled with media from the respective condition. The collected media and wash were centrifuged at 300 RCF for 5 min. After centrifugation supernatant was collected into a new 15 mL conical. The supernatant was then centrifuged at 2,140 RCF for 5 min. The supernatant was then aspirated off and the pellet was resuspended in 100 µl of PBS (6-well) or stain buffer (T-25). MSCs were collected using 1.5 mL 0.25% trypsin–EDTA (1x) (Gibco, cat#: 25200-056) for 3–5 min at 37 °C, 5% CO_2_. Following incubation, trypsin was neutralized with 1.5 mL 10% total DMEM. Cell suspensions were collected and centrifuged at 500 RCF for 5 min. The resulting cell pellet was combined with the pellet created from the media and PBS washes.

For analysis via fluorescent microscopy, cultures in 6-well plates were stained 30 min prior to cell collection. For analysis via flow cytometry, MSC cultures were first harvested from T-25 flasks then labeled. Staining methods are described in the following sections.

## MSC viability assessment via fluorescent microscopy

At each time point, 4.02 µM of Calcein AM (Invitrogen, cat # 65-0853-39) and either 3.61 µM (cell-impermeant) or 36.1 µM (cell-permeant) 4’,6’-diamidino-2-phenylindole (DAPI) (Sigma Aldrich, cat # 10236276001) were added to each well and incubated at 37˚C, 5% CO_2_ for 30 min (Additional file 1: Figure S1). The lower, cell-impermeant levels of DAPI were used for MSC imaging in culture. The concentration of DAPI was increased to cell-permeant concentrations for scoring viable MSCs and allow for the exclusion of anucleate cell debris. The concentration-dependent permeability of DAPI for live cell imaging was ideal for our microscopy assays compared to alternative impermeant DNA dyes (e.g. propidium iodide) and does not overlap in excitation or emission spectrum used for calcein-AM imaging, minimizing background. After completing the staining and cell collection protocol (above), 10 µl of each HCT and RBC_rel_ condition was scored on a Neubauer counting chamber (Hausser Scientific, cat # 3310 V) for MSC viability. Calcein^+^/DAPI^+^ MSCs were scored as viable whereas calcein^−^/DAPI^+^ were scored as dead. Cultures and viability scoring were performed on an AMG EVO FL inverted digital microscope using light cubes: DAPI excitation 357 nm/44BP excitation; 447 nm/60BP emission and GFP 470 nm/22BP excitation; 510 nm/42BP emission.

### Staining and analysis of viable, apoptotic and necrotic MSC populations via flow cytometry

All flow cytometry was performed using a Beckman Coulter CytoFLEX S equipped with a 488 nm laser and 525 nm/40BP, 585 nm/42BP, 690 nm/50BP filters and a 638 nm laser with a 660 nm/20BP filter. Cell suspensions and supernatants were stained using CD90-conjugated to FITC (Biolegend, cat. #328108) to gate for MSCs, annexin-V-conjugated (AV) to APC (Biolegend, cat. #640920) to detect apoptotic cells and 7-aminoactinomycin D (7AAD) viability stain (Invitrogen, cat. #00-6993-50) to detect necrotic cells in AV Binding Buffer (Biolegend, cat. #422201) with color compensation applied. A master stock staining solution was created by combining 30 µl FBS, 570 µl AV Binding Buffer, 37.5 µl CD90, 37.5 µl AV, and 75 µl 7AAD. MSC pellets isolated from T-25 culture was resuspended in 100 µl staining buffer, merged with pellets derived from media and washes, and incubated at room temperature and protected from light for 30 min.

Initial gating criteria and confirmation of apoptotic MSC via staining for CD90^+^, AV^+^ and/or 7AAD^+^ was achieved via exposure to 38.2 nM H_2_O_2_ over a 24-h time course (Fig. 2a). Gating for necrotic MSCs (CD90^+^, AV^−^, 7AAD^+^) was determined via exposure to 5 µM doxorubicin over a 40-min time course (Fig. 2a). In experimental series for HCT and RBC_rel_, the 0% HCT/ RBC_rel_ control was gated based on the respective isotype and dye control at each time point, revealing the baseline for viable, apoptotic and necrotic MSC populations. For each condition (% HCT or % RBC_rel_), time-point and experimental run, gates were established based on the 0% control and the distribution of percentage viable (CD90^+^/AV^−^/7AAD^−^), apoptotic (CD90^+^/AV^+^/7AAD^±^) and necrotic (CD90^+^/AV^−^/7AAD^+^) MSCs in all experimental HCT and RBC_rel_ conditions were reported.

### Normalization of MSC viability based on count

Reporting MSC viability based on the percentage of a population does not capture diminished MSC counts resulting from degradative/disintegration processes associated with apoptosis and necrosis. To account for the potential changes in viable MSCs concentrations relative to the control, MSC viability in HCT and RBC_rel_ conditions were normalized based on the CD90^+^/AV^−^/7AAD^−^ counts per microliter, multiplied by the live percentage and divided by the same figures for the 0% control: ((MSC Count/µl_condition_)(% Viable_condition_))/((MSC Count/µl_control_)(% Viable_control_)). The resulting figure reflects a normalized quantitation of viable MSCs, shown as a percentage relative to the control.

### Lipid peroxidation assay and imaging

Bone marrow derived human MSCs were seeded at 5000 cells per well in a 12-well plate (passage 3); low density seeding was selected to allow clear visualization of individual cells during imaging and minimize background. Elevated HCT conditions (10%, 20% and 40% HCT) were incubated with MSC cultures for 48 h; untreated controls were held in total media. Fluorescent staining was performed using a lipid peroxidation kit for live cell analysis (Thermo Scientific, cat. #C10445) according to manufacturer’s instructions. Unlabeled, untreated/negative and positive (cumene hydroperoxide) controls were established. Images were captured via AMG EVO FL inverted digital microscope using light cubes: GFP 470 nm/22BP excitation; 510 nm/42BP emission and Texas Red 585/29nmBP excitation; 628/32 nm emission and overlayed with a phase contrast image. Green fluorescence indicates lipid peroxidation whereas red fluorescence indicates normal lipids. All images were captured using a set light intensity for each light cube/channel and merged. Experiment was repeated twice to confirm observations.

#### Statistical analysis

Data was analyzed in GraphPad Prism version 8.4.3 using one-way ANOVA followed by a Dunnett’s multiple comparison test to determine levels of significance with a 95% confidence interval; levels of significant are shown as *p* < 0.05 and *p* < 0.01. Data from all HCT and RBC_rel_ conditions for MSC viability, apoptosis and necrosis were tested against the respective 0% control in multiple comparison tests.

## Results

### Qualitative assessment of MSC viability with relation to HCT in vitro

**Fig. 1:**
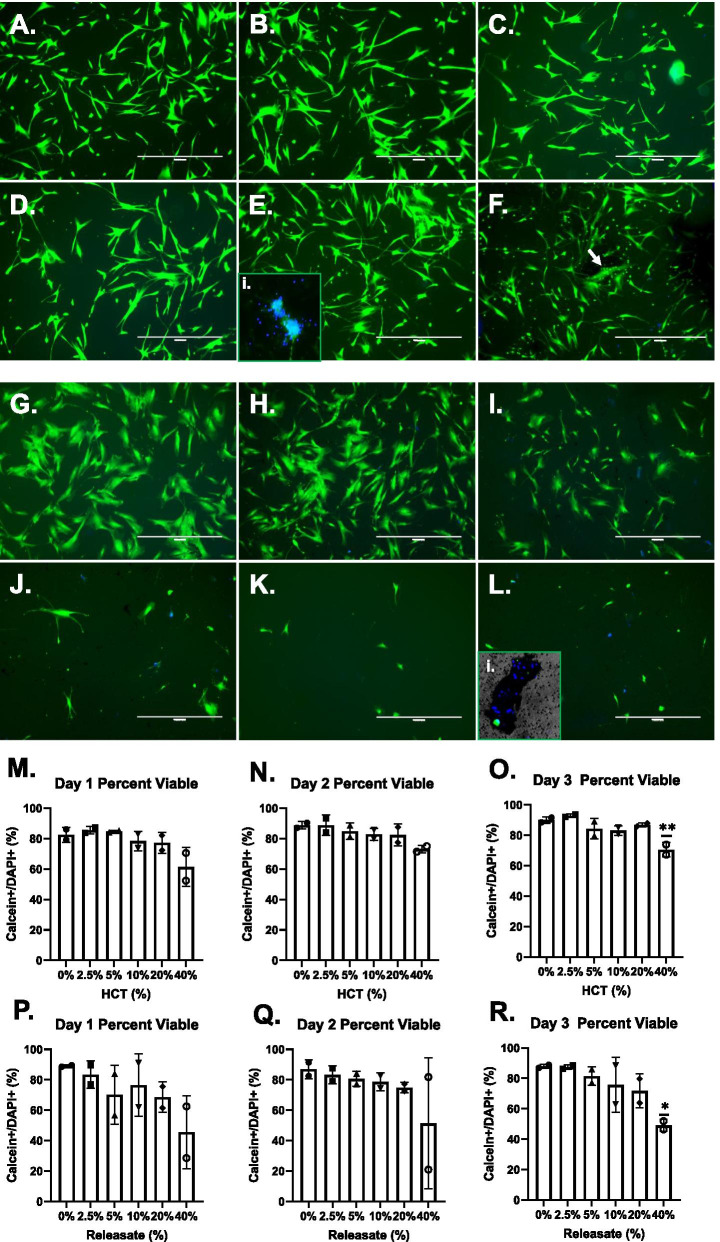
Fluorescent imaging of MSCs in HCTs at Day 1 (**A**–**F**), Day 3 (**G**–**L**) and quantitation of MSC viability Day 1–3 for HCT (**M**–**O**) and RBC_rel_ (**P**–**R**). MSCs in culture were stained with calcein-AM (green, live) and DAPI (blue, dead) and imaged prior to harvesting for viability analysis. Day 1 (**A**). 0% HCT control (**B**). 2.5% HCT (**C**). 5% HCT (**D**). 10% HCT (**E**). 20% HCT, (**E**)(i). 20% HCT with fragmentation of nuclei (subpanel is enlarged, not to scale) (**F**). 40% HCT (arrow shows microparticle formation from MSC). Day 3 (**G**). 0% HCT control (**H**). 2.5% HCT (**I**). 5% HCT (**J**). 10% HCT (**K**). 20% HCT (**L**). 40% HCT, (**L**)(i). 40% HCT with RBC aggregate containing non-viable MSCs (subpanel is enlarged, not to scale). For all micrographs: Scale bar = 860 microns; Total magnification = 104x (excluding **E**)(i). and (**L**)(i).). Quantitation of viable MSCs via fluorescent microscopy in HCT and RBC_rel_ conditions. Viable MSCs in HCT conditions at (**M**) Day 1, (**N**) Day 2 and (**O**) Day 3. Viable MSCs in RBC_rel_ conditions at (**P**) Day 1, (**Q**) Day 2 and (**R**) Day 3. Error bars represent the standard deviation of the data set. Levels of significance: *p < 0.05, **p < 0.01

Individual MSC cultures established in 6-well plates received HCTs of 0%, 2.5%, 5%, 10%, 20% and 40% respectively. At Days 1, 2 and 3, cultures were imaged for viability. At Day 1, with higher HCTs, MSC morphology appeared to be altered (Fig. 1A–F), showing an elongated appearance, evidence of nucleus fragmentation and calcein^+^ microparticle formation (Fig. 1E(i) and F respectively). By Day 3, MSC density and viability were clearly reduced (Fig. 1G–L), MSC morphology was abnormal and clusters of nuclei were encapsulated in RBC aggregates were observed (Fig. 1L(i)). MSC cultures exposed to RBC_rel_ derived from the respective HCTs were also imaged. Similar to the HCT cultures, the RBC_rel_ -treated MSCs showed increasing death and fewer MSCs compared to the 0% control (data not shown).

### Quantitation of MSC viability via fluorescent microscopy in variable HCT and RBC_rel_ conditions

Collection of adhered MSCs from HCT and RBC_rel_ cultures were quantitated via fluorescent microscopy. MSCs were stained with calcein to detect metabolically active cells as well as a high concentration of DAPI (10 µg/mL) to detect the presence of DNA for live cell imaging (viable MSCs are calcein^+^/DAPI^+^, dead are calcein^−^/DAPI^+^). Live MSC counts were not significantly reduced between any HCT or RBC_rel_ condition at Day 1 or Day 2 (Fig. 1M,N,P,Q). At Day 3, the 40% HCT showed a significant reduction in MSC viability compared to the 0% HCT control (*p* < 0.01) (Fig. 1O). In RBC_rel_ treatments, a significant reduction in viable MSCs was also observed in the 40% RBC_rel_ compared to the 0% RBC_rel_ (*p* < 0.05) (Fig. 1R). This scoring method resulted in several small, calcein^+^/DAPI^+^ particles which may be indicative of apoptotic bodies though, could not be appropriately discriminated from intact MSCs based on size alone.

### Increasing HCT and RBC_rel_ conditions reduces MSC viability

**Fig. 2:**
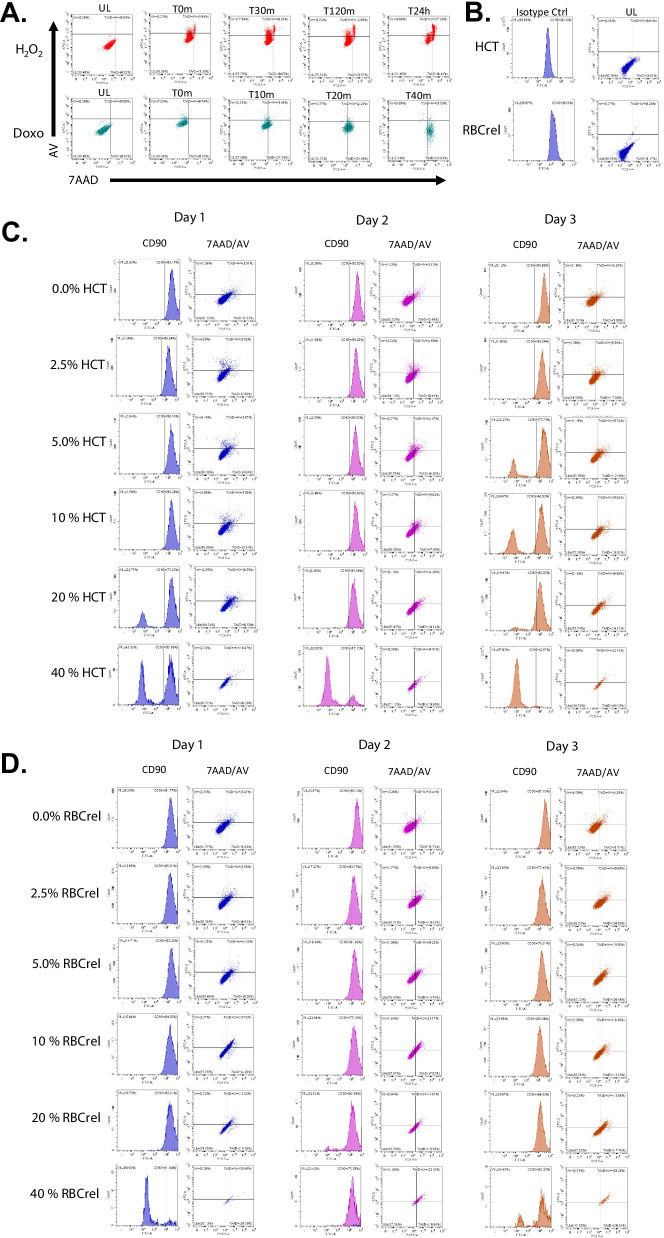
Staining, gating and analysis of MSCs via CD90, AV and 7AAD. **a** Controls for apoptosis and necrosis via H_2_O_2_ and doxorubicin, respectively. **b** Untreated MSCs gated via FITC-isotype control for CD90 gating (histogram) and unlabeled for AV and 7AAD (complimenting the axes in “**a**”) for the following HCT and RBC_rel_ data sets. **c** CD90-labeled MSC health status in HCT conditions Day 1–3. **d** CD90-labeled MSC health status in RBC_rel_ conditions Day 1–3. Dot plot quadrants for **c** and **d** are identical to the labeled controls in (**a**) and are as follows: viable (AV-/7AAD-) are in the lower left quadrant, AV + /7AAD- are upper left quadrant, AV + /7AAD + upper right and AV-/7AAD + are lower right quadrant

To appropriately discriminate viable, apoptotic and necrotic MSCs from apoptotic bodies (cell fragments), HCT and RBC_rel_ conditions were repeated in larger cultures and cells were labeled for CD90, AV and 7AAD at Days 1, 2 and 3 and analyzed via flow cytometry (Fig. 2).

**Fig. 3:**
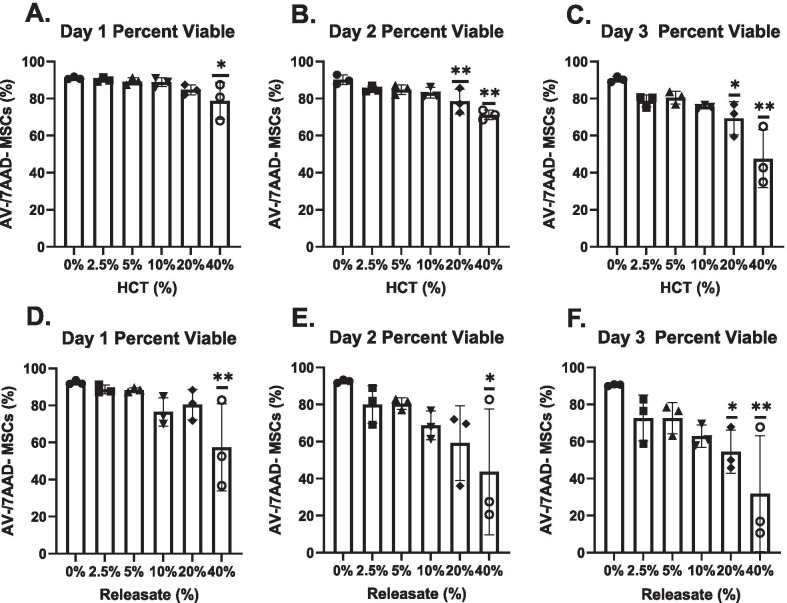
Percentage of viable MSCs via flow cytometry in HCT and RBC_rel_ conditions. MSCs stained with CD90, annexin-V (AV) and 7AAD where CD90 + /AV-/7AAD- are viable MSCs. Viable MSCs in HCT conditions at **a** Day 1, **b** Day 2 and **c** Day 3. Viable MSCs in RBC_rel_ conditions at **d** Day 1, **e** Day 2 and **f** Day 3. Error bars represent the standard deviation of the data set. Levels of significance: *p < 0.05, **p < 0.01

At Day 1, the percentage of viable MSCs in 40% HCT conditions were significantly reduced compared to the 0% control (Fig. 3a) (*p* < 0.05). At Day 2, both the 20% and 40% HCT differed significantly from the 0% control (*p* < 0.01) (Fig. 3b). The percentage of viable MSCs in the 20% and 40% HCT conditions remained significantly lower at Day 3 compared to the control (*p* < 0.05 and *p* < 0.01 respectively) (Fig. 3c).

In RBC_rel_ at Day 1 and Day 2, the percent viable MSCs cultured in 40% RBC_rel_ was significantly reduced compared to the 0% control (*p* < 0.01 and *p* < 0.05 respectively) (Fig. 3d, e). Respective to the control at Day 3, MSCs cultured in 20% and 40% RBC_rel_ conditions contained a significantly lower viable population (*p* < 0.05 and *p* < 0.01 respectively) (Fig. 3f).

### Early necrosis in high HCT and RBC_rel_ is followed by apoptosis in MSCs

**Fig. 4:**
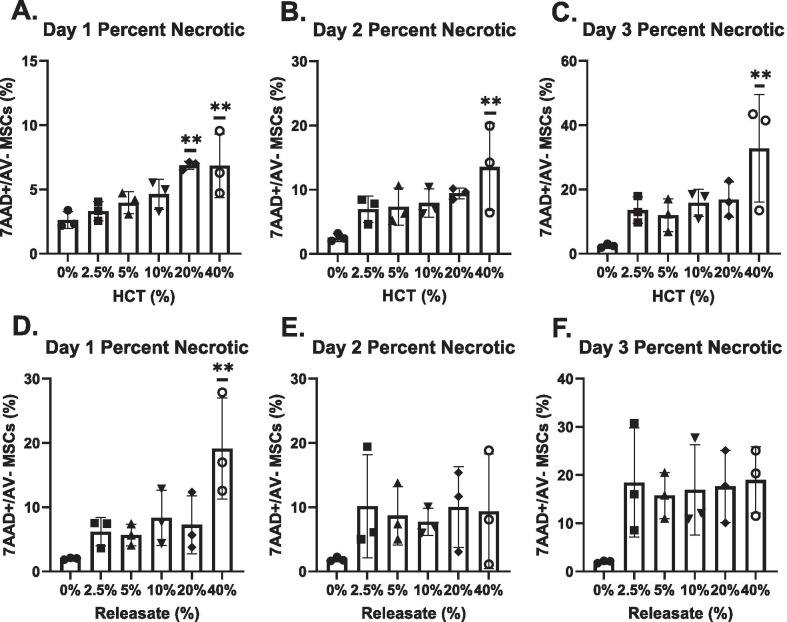
Percentage of necrotic MSCs via flow cytometry in HCT and RBC_rel_ conditions. MSCs stained with CD90, annexin-V (AV) and 7AAD where CD90 + /AV-/7AAD + are necrotic MSCs. Necrotic MSCs in HCT conditions at (**a**) Day 1, (**b**) Day 2 and (**c**) Day 3. Necrotic MSCs in RBC_rel_ conditions at (**d**) Day 1, (**e**) Day 2 and (**f**) Day 3. Error bars represent the standard deviation of the data set. Levels of significance: *p < 0.05, **p < 0.01

Necrotic MSCs at Day 1 in the 20% and 40% HCT conditions were significantly higher compared to the 0% HCT condition (*p* < 0.01 for each) (Fig. 4a). At Days 2 and 3, necrotic MSCs in the 40% HCT condition were significantly elevated compared to the 0% HCT control (*p* < 0.01 at each timepoint) (Fig. 4b, c). In RBC_rel_ treatments, the percentage of necrotic MSCs was only significantly elevated over the control in the 40% RBC_rel_ condition at Day 1 (Fig. 4d) (*p* < 0.01).

**Fig. 5:**
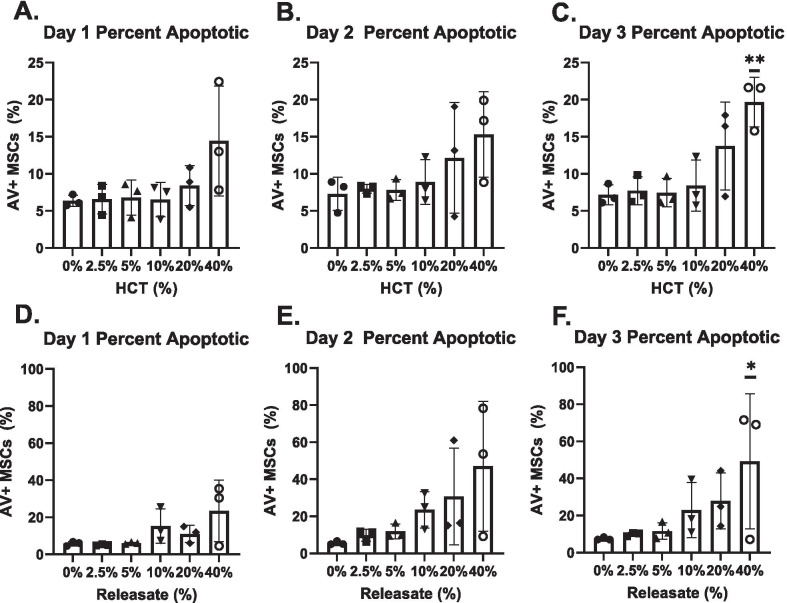
Percentage of apoptotic MSCs via flow cytometry in HCT and RBC_rel_ conditions. MSCs stained with CD90, annexin-V (AV) and 7AAD where CD90 + AV + / 7AAD ± are apoptotic MSCs. Apoptotic MSCs in HCT conditions at (**a**). Day 1 (**b**). Day 2 and (**c**). Day 3. Apoptotic MSCs in RBC_rel_ conditions at (**d**). Day 1 (**e**). Day 2 and (**f**). Day 3. Error bars represent the standard deviation of the data set. Levels of significance: *p < 0.05, **p < 0.01

In both HCT and RBC_rel_ conditions, the percentage of apoptotic MSCs did not differ between the treatment groups and the control at Day 1 and Day 2. The percentage of apoptotic MSCs in both 40% HCT and 40% RBC_rel_ conditions reached significance at Day 3 (*p* < 0.01 and *p* < 0.05 respectively) (Fig. 5c, f).

### HCT and RBC_rel_ significantly reduce total viable MSCs

Dynamic changes in MSC viability, apoptosis and necrosis with increasing HCT and RBC_rel_ concentrations suggests that cell death is occurring rapidly. While percentages of viable, apoptotic and necrotic MSCs is informative, it does not account for the loss of MSCs quantitatively following necrotic and apoptotic processes (observed in Fig. 1). MSC viability normalized to cell count was used to determine the relative differences in viable MSC quantities in each condition.

**Fig. 6:**
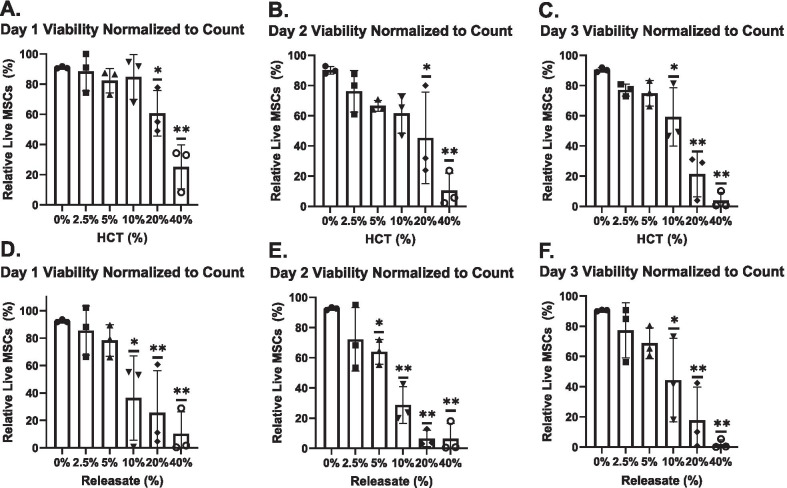
Normalized quantitative MSC viability via flow cytometry in HCT and RBC_rel_ conditions. MSCs stained with CD90, annexin-V (AV) and 7AAD where CD90 + /AV-/7AAD- are viable; percentages derived from viable counts in each condition relative to the control. Viable MSCs in HCT conditions at **a** Day 1, **b** Day 2 and **c** Day 3. Viable MSCs in RBC_rel_ conditions at **d** Day 1, **e** Day 2 and **f** Day 3. Error bars represent the standard deviation of the data set. Levels of significance: *p < 0.05, **p < 0.01

At Day 1 and Day 2, the relative number of viable cells in 20% and 40% HCT differed from the control (*p* < 0.05 and *p* < 0.01 respectively at each time point) (Fig. 6a, b). At Day 3, quantitative viable MSC populations in the 10%, 20% and 40% HCT conditions were significantly reduced compared to 0% HCT condition (*p* < 0.05, *p* < 0.01, *p* < 0.01 respectively) (Fig. 6c).

In RBC_rel_ treatments, viable MSCs quantitatively were significantly reduced in 10%, 20% and 40% conditions at Day 1 compared to the control (*p* < 0.05, *p* < 0.01, *p* < 0.01 respectively) (Fig. 6d). Relative to the 0% control at Day 2, viable MSCs were significantly reduced in 5% (*p* < 0.05), 10% (*p* < 0.01), 20% (*p* < 0.01) and 40% (*p* < 0.01) RBC_rel_ conditions (Fig. 6e). At Day 3, significant reductions in viable MSCs were observed in 10%, 20% and 40% RBC_rel_ conditions relative to the control at Day 3 (*p* < 0.05, *p* < 0.01, *p* < 0.01 respectively) (Fig. 6f).

### RBCs induce lipid peroxidation in MSCs

**Fig. 7:**
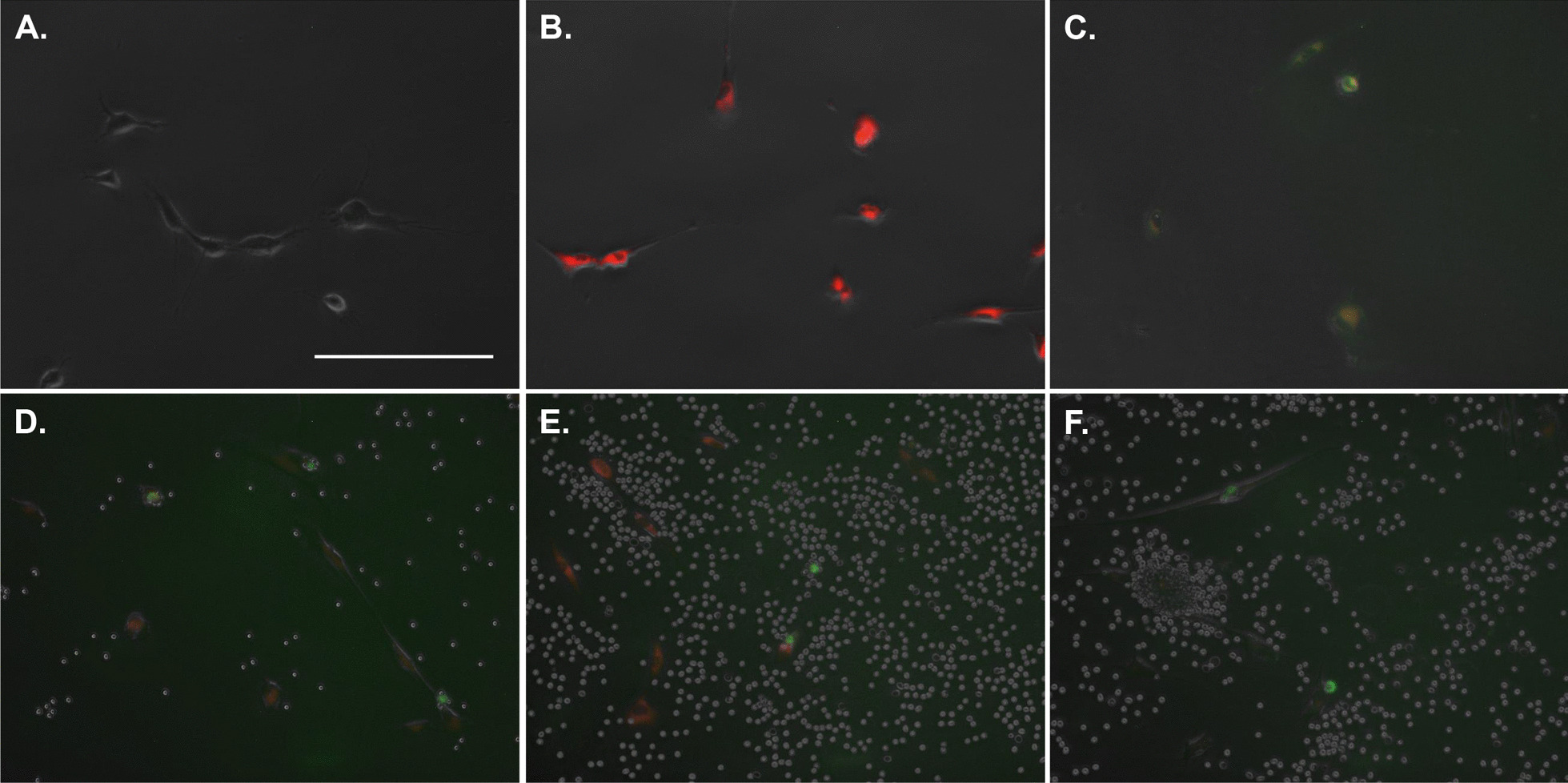
Fluorescent imaging of lipid peroxidation in MSCs exposed to high HCTs. **a** Unstained control, **b** negative control staining for normal lipids (no peroxidation, red), **c** positive lipid peroxidation control, MSCs treated for 2 h with cumene hydroperoxide (green signal). MSCs incubated for 48 h prior to staining with **d** 10% HCT, **e** 20% HCT and **f** 40% HCT show evidence of lipid peroxidation (green) and cell detachment (rounded signals) and dampening of red fluorescence. Unstained cells in **d**–**f** are residual RBCs post-washing. Scale bar = 200 microns. For all micrographs: total magnification = 520×

The reduction in viable MSC counts suggests cell detachment and the possibility of a unique mode of cell death, ferroptosis, where lipid peroxidation is an indicator of this mode of cell death (28, 29). To assess this possibility, MSCs were incubated with 10%, 20% and 40% HCT for 48 h and fluorescently imaged for evidence of lipid peroxidation (Fig. 7). The unlabeled control showed no background fluorescence whereas the untreated control was strongly positive (red) for undamaged lipids (no lipid peroxidation) and the cumene hydroperoxide control showed the appropriate shift to green fluorescence, indicating lipid peroxidation (Fig. 7a–c). In all HCT conditions, lipid peroxidation was observed along with a reduction in red fluorescent signal relative to the untreated control (Fig. 7d–f). Where lipid peroxidation evident via fluorescence, cell detachment (rounded morphology) was observed concurrently.

## Discussion

The objective when producing BMC from BMA is to concentrate and purify the NC and MSC populations, which is achieved via centrifugation and collection of the buffy coat (nucleated cells) at the plasma/RBC interface. Acquisition of the buffy coat often includes “dipping” into the RBC interface to maximize NC and MSC yields, elevating the HCT [14]. Alternatively, others propose the direct delivery of unprocessed BMA in order to minimize NC and MSC loss during processing [28]. Traditionally, MSC quantitation is determined via CFU-f in culture over a 7–14 day time course [29] and is a common metric to determine BMA/BMC quality. This in vitro method presents numerous confounding factors which artificially skew and minimize the impact of RBCs on MSC health and is not representative of BMA/BMC performance in vivo. As an example, when plating for CFU-fs, a small aliquot of BMA/BMC sample, on the order of microliters, is added to several milliliters of culture media in a culture vessel, resulting in significant sample dilution and the associated HCT. Additionally, some protocols utilized RBC lysis buffers prior to plating BMC for CFU-f analysis, eliminating the impact of the RBCs on MSC growth [29, 30]. Furthermore, cultures are typically washed within 24–72 h, removing nonadherent cells, including white blood cells (with the exception of monocytes) and RBCs [31]. Taken together, the CFU-f assay may be adequate for quantitation of MSCs in a specimen but, may also lack translational value as it does not emulate the survival or health of BMA/BMC-derived MSCs under in vivo conditions where dilution of RBCs is minimal. To date, a single article has directly pointed out that RBC content appears to impact CFU-f frequency [32] though, with no further investigation into the influence of RBCs on MSC health and did not offer a plausible explanation for this observation. While others have speculated the potential negative impact of RBCs in BMC products (and other orthobiologics) [24], no in vitro research exists to support or refute this possibility.

Traditionally, the anucleate, organelle-free RBC has been considered to be relatively inactive with respect to cytokine secretion and has not been recognized as a factor in BMA/BMC therapeutics. Recently, an elegant study by Karsten et al. provided important insight to the RBC secretome, intracellular and surface cytokine reservoirs which may have significant implications in pro-inflammatory processes [27]. Importantly, they demonstrate a significant reduction in IL-1β, IL-6, IL-8 and TNF-α in RBC conditioned media (RBC_rel_) derived from RBCs which were washed multiple times in PBS compared to the control RBC condition (1 × PBS wash). Further, the results presented by Karsten et al. offer a rationale for previously recorded complications and pathologies associated with unwashed RBC packs post-transplantation compared to washed RBC packs, suggesting that the adverse effects may be linked to red blood cell-dependent release of inflammatory proteins/factors.

The present investigation sought to determine if intact red blood cells and their releasates have an effect of MSC health over a short time course. Fresh red blood cells used to produce the various HCT conditions were derived from the bone marrow to simulate BMC HCT conditions across a broad spectrum, including unprocessed BMA (40% HCT condition) which resembles the final product resulting from non-centrifugation BMA collection devices [28]. RBC_rels_ were produced by aging HCTs at 37˚C for 72 h, resulting in RBC byproduct accumulation in the media and mirrors the method used by others to produce RBC conditioned plasma [27, 33]. The RBC-depleted releasates used in this study are intended to simulate red blood cell secretory-, degradation- and eryptosis-related factors in days following an injection in vivo. Taken together, we are able to contrast the effect of intact RBCs (HCT) and the respective releasates (RBC_rel_) on MSC health.

Our initial assay utilized fluorescent microscopy to image and score MSC viability, where quantitation utilized high concentrations of DAPI to permeate cell membranes and verify the presence of a nucleus in the cell in conjunction with calcein. As red blood cells show weak-to-no signal from calcein due to its interaction with iron ions (Fe^2+^ and Fe^3+^) and signal quenching [34], this allows the discrimination of MSCs from RBCs. Further, calcein^+^ particles lacking DAPI signal were not counted as an MSC. While there were clear differences in the adhered MSC morphology and MSC viability at higher HCT and RBC_rel_ conditions compared to the 0% control, it was not possible to absolutely contrast MSCs from potential apoptotic bodies, which can reach 5 µm and typically contain small amounts of fragmented DNA and confound our quantitation method [35]. Furthermore, initial imaging of wells showed higher densities of DAPI foci in what appear to be RBC aggregates, which were not successfully recovered following washes and scoring. To address the issue of quantifying viable MSCs and contrast from apoptosis (AV^+^) and necrosing (7AAD^+^) MSCs, larger cultures were utilized and analyzed via flow cytometry to detect the respective state of the MSC.

Flow cytometry revealed the rapid changes in the percentage of viable, necrotic and apoptotic MSCs in higher HCT and RBC_rel_ conditions compared to the 0% control over the short time course. Regarding viability, the results mirror those of the fluorescent microscopy assay though, with higher resolution allowing for the exclusion of apoptotic bodies. Our results demonstrate that as HCT or RBC_rel_ concentrations increase, the percentage of viable MSCs decreases and is accompanied by necrosis and apoptosis. In addition, it appears that MSCs initially undergo necrosis when exposed to high HCTs and RBC_rel_, followed by apoptosis concurrent with necrosis at Day 3. However, the percentage of viable, apoptotic and necrotic MSCs offer only “snap-shots” in time and are not quantitative. These percentages of the overall MSC population at each time point do not account for quantitative loss of MSCs resulting from disintegration processes associated with necrosis and apoptosis. This is explained by the changes in cell integrity during necrosis and apoptosis. Cells which undergo necrosis undergo disintegration phases [36], leading to a complex series of cellular breakdown and result in debris which would not meet our criteria for detecting intact CD90^+^ cells within the MSC established gate and are not included in the represented percentage. Further, those which initiate apoptosis undergo extensive “blebbing”, forming numerous apoptotic bodies ranging from ~ 0.05–5.0 µm in size [35]. These entities, while retaining the CD90^+^ marker and label positive for AV, are smaller than the intact MSCs and do not reside in the appropriate gate based on the cell size and intracellular complexity via flow cytometry (forward scatter and side scatter respectively). As a result, apoptotic bodies are excluded from the apoptotic MSC population. In the case of both necrosis and apoptosis, the cumulative number of MSCs are reduced though, are not accurately reflected when reported as a general percentage.

To address this conflict, viability was normalized. This was achieved by determining the viable MSC count per microliter for each condition and shown as a percentage relative to the control. In this way, the reduction in overall viable MSCs at each time point compared to the respective control was captured. The results revealed the sharp contrast of HCT and RBC_rel_ conditions ≥ 10% compared to the control and supports the concept that MSC death and deterioration is ongoing over the time course leading to substantial reductions in the total MSC population. In addition, this data supports our observations using fluorescent microscopy where MSC density in the cultures were clearly diminished by Day 3 (Fig. 2).

The precise mechanism(s) by which HCT/RBCrel induces MSC necrosis and apoptosis remains unclear. While both modes of cell death were observed, there are likely multiple causative factors, including but not limited to those described by Karsten et al. The possibility of increasing levels of free heme/hemin in the media as the result of RBC decay led us to investigate the possibility of ferroptosis, a unique iron-dependent mechanism of cell death which is closely associated with lipid peroxidation [37, 38]. Our observations demonstrate that lipid peroxidation does in fact occur to some extent in conditions containing ≥ 10% HCT, suggesting ferroptosis as an additional mode of RBC-induced MSC death. Ferroptosis has only recently been described and has a unique intracellular pathway resulting in cell death, which is distinct from traditional apoptosis and necrosis and is often characterized by cell detachment concurrent with death [37]. Further, cells undergoing ferroptosis do not produce hallmarks of normal apoptosis such as the formation of apoptotic bodies and cell shrinkage [39] though, stain positive for phosphatidylserine via annexin V [40]. Similarly, cells undergoing ferroptosis do not present typical characteristics of necrosis such as cell and organelle swelling and cell membrane rupture/permeability [39] though, the compromised cell membrane allows for DNA-staining with impermeant dyes [40]. However, it is not probable that ferroptosis is the exclusive mode of cell death resulting from RBCs, as evidence of cell fragmentation was apparent via fluorescent microscopy and flow cytometry detected small CD90 + /annexin-V + particles outside of the established MSC gate (not represented in the data sets), indicative of canonical apoptosis induced by RBC-derived factor(s).

The HCT conditions represent events which occur with regards to the MSC at early time points following in vivo injection of BMC, while RBC_rel_ accounts for latter events which are associated with deterioration, degradation and release of various cytokines and hemoglobin along with iron ions. While we did not observe substantial differences between HCT and RBC_rel_ conditions, our results indicate that both have negative effects on MSCs in vitro. Translationally, this data is highly suggestive that the deleterious effects of RBCs and their releasates on MSCs occurs rapidly and may have a prolonged impact in vivo following RBC deterioration, which is of high importance in regions with low vascularity where debris is not efficiently removed. In addition, CFU-f quantitation on BMC products should document the HCT of the product, as the high dilution factor of BMA/BMC in culture overlooks the impact of HCT in the therapeutic and may lead to non-translational CFU-f metrics. Ultimately, higher CFU-f values associated with high HCT may mean little if in vivo, > 80% of the MSCs are expected to undergo necrosis/apoptosis within days.

While others have shown that RBCs can have a negative impact on cell-types associated with orthopedic tissues [25, 26], this study is the first to demonstrate deterioration in MSC health following exposure to HCT and RBC_rel_ conditions. Our results strongly suggest that the production of BMC or BMA products with an HCT > 10% may lead to significant MSC death within days once injected in vivo, limiting the efficacy of the intended treatment. Furthermore, the results provide a potential explanation for the inconsistencies in the literature regarding the efficacy of BMC treatments for orthopedic conditions and RBC quantitation should be routinely reported in clinical studies evaluating BMC as a treatment option. Our findings have significant implications beyond the field of orthopedics and further the findings of others where RBC contamination poses deleterious effects on bone marrow mononuclear cell therapies following myocardial infarction and stroke [41, 42]. Ultimately, clinicians should aim to produce BMC products with HCTs below 10% and HCT should be considered an indicator of product quality.

## Conclusions

HCT and RBC_rel_ induce bone marrow-derived MSC death via necrosis and apoptosis, rapidly reducing viable MSC counts over a 3-day time course. These findings demonstrate that both RBCs and the byproducts of their deterioration are toxic to MSCs. HCT should be carefully controlled in orthobiologics with a threshold of < 10% or, approximately a > fourfold-reduction of RBCs in BMC relative to the original BMA to avoid compromising the integrity of the therapy and optimize clinical outcomes. Regarding future clinical studies, HCT and/or RBC count should be reported along with cell distribution data as it may directly influence patient response.

## Supplementary Information

**Additional file 1**.** Figure S1**: Live Cell Imaging using DAPI at cell impermeant (3.6059 µM; 1 µg/mL) and permeant concentrations (36.059 µM; 10 µg/mL) with calcein-AM. Total Magnification = 260×. Scale Bar = 400 µm.

## Data Availability

The datasets used and/or analyzed during the current study are available from the corresponding author on reasonable request.

## Abbreviations

PRP: Platelet-rich plasma
BMA: Bone marrow aspirate
BMC: Bone marrow aspirate concentrate
MSK: Musculoskeletal
MSC: Mesenchymal stem cell
TNC: Total nucleated cell count
NC: Nucleated cell
CFU-f: Colony forming unit fibroblasts
HCT: Hematocrit
CAR: CXCL12-abundant reticular cells
RBC: Red blood cell
RBC_rel_: Red blood cell releasate
RCF: Relative centrifugal force
FBS: Fetal bovine serum
PBS: Phosphate buffered saline
Calcein/calcein-AM: Calcein acetoxymethyl ester
DAPI: 4’,6’-diamidino-2-phenylindole
AV: Annexin-V
7AAD: 7-Aminoactinomycin
ANOVA: Analysis of variance
