# Supplementary figures and images for: Red blood cells and their releasates compromise bone marrow-derived human mesenchymal stem/stromal cell survival in vitro
Source: Stem Cell Res Ther. 2021 Oct 21;12:547. doi: 10.1186/s13287-021-02610-4 (PMC8529765; doi:10.1186/s13287-021-02610-4)

## Slide 1
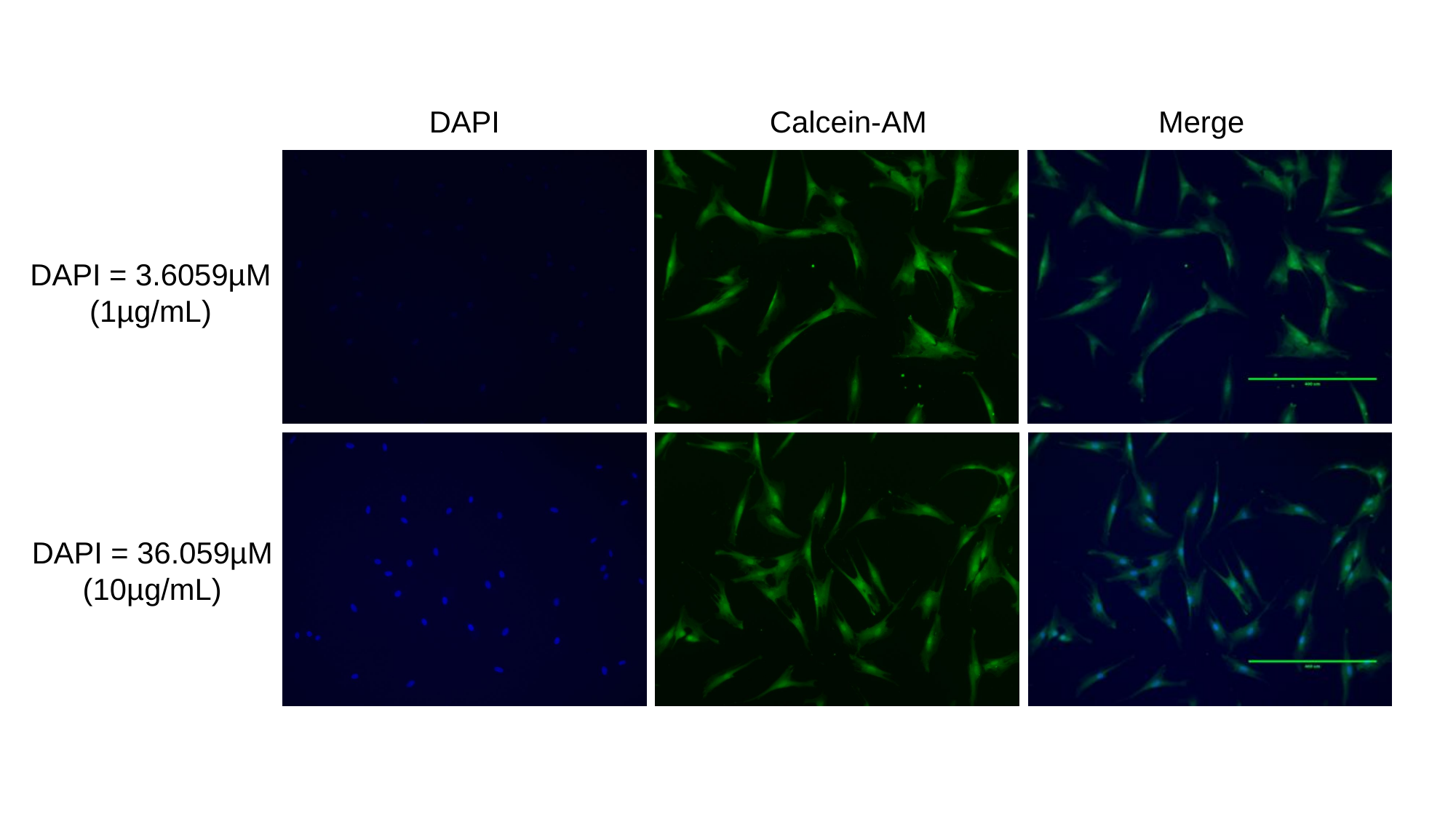

DAPI
Calcein-AM
Merge
DAPI = 3.6059µM
(1µg/mL)
DAPI = 36.059µM
(10µg/mL)

Supplement: Supplementary file 1 — Additional file 1. Figure S1: Live Cell Imaging using DAPI at cell impermeant (3.6059 µM; 1 µg/mL) and permeant concentrations (36.059 µM; 10 µg/mL) with calcein-AM. Total Magnification = 260×. Scale Bar = 400 µm. [file 13287_2021_2610_MOESM1_ESM.pptx]
